# A32 ARTIFICIAL INTELLIGENCE USE IN DIAGNOSIS & MONITORING OF INFLAMMATORY BOWEL DISEASE: A SCOPING REVIEW

**DOI:** 10.1093/jcag/gwae059.032

**Published:** 2025-02-10

**Authors:** G Malik, M Chavannes, M F Byrne, M Dolinger, S Sagami, K Novak

**Affiliations:** Gastroenterology and Hepatology, University of Calgary Cumming School of Medicine, Calgary, AB, Canada; Children’s Hospital Los Angeles, Los Angeles, CA; The University of British Columbia, Vancouver, BC, Canada; Icahn School of Medicine at Mount Sinai, New York, NY; Kitasato Daigaku Medical Center, Kitamoto, Saitama, Japan; Gastroenterology and Hepatology, University of Calgary Cumming School of Medicine, Calgary, AB, Canada

## Abstract

**Background:**

Inflammatory bowel diseases (IBD) are a family of immune-mediated conditions, which are increasing in incidence and prevalence worldwide. Assessment of IBD is done through endoscopy, video capsule endoscopy (VCE), histology, and various imaging modalities including ultrasound (US), computed tomography (CT), and magnetic resonance imaging (MRI). Considering the increasing complexities in the assessment of IBD, artificial intelligence (AI) is an important adjunct with potential to enhance diagnosis, drug response and prediction of disease course

**Aims:**

We conducted a scoping review to assess AI in diagnosis, monitoring, and prognostication of patients with IBD, to aid in identification of gaps in knowledge to guide future research endeavors.

**Methods:**

The scoping review protocol was adapted from the recommendations laid out by the Preferred Reporting Items for Systematic Reviews and Meta-Analysis - Scoping Review Extension (PRISMA-ScR). Electronic databases used in the literature search included MEDLINE, EMBASE, the Cochrane Library, Cumulative Index to Nursing and Allied Health Literature, and Engineering Village. Two reviewers independently screened the abstracts and titles first before performing full text review. A third review resolved any conflict where needed. All study types were included, and data extraction utilized Covidence. Studies were categorized based on the assessment modality, then themes including, diagnosis, grading activity, prognosis, and monitoring.

**Results:**

A total of 140 studies were included in the final scoping review. The largest number of studies involved endoscopy at 72 (51%) citations, followed by VCE, histology, MRI, CT, and US at 30 (21%), 18 (13%), 13 (9%), 6 (4%), and 1 (0.7%) citation(s), respectively. When looking at themes, most endoscopy studies examined disease activity (65%) while diagnosis was the most common theme in VCE, MRI and CT (77%, 69% and 83%, respectively). Histologic studies focused on prognosis (89%) and the single US study evaluated both diagnosis and prognosis concomitantly. Amongst all the investigative modalities examined, monitoring of IBD was the least studied theme. Peak performance of AI models for grading disease activity during endoscopy was 98.7% compared to human clinicians with less variability observed.

**Conclusions:**

With IBD diagnosis and assessment becoming increasingly complex, AI may be a useful adjunctive tool across multiple modalities. Evaluation of use of AI in US is lacking, despite gaining interest in non-invasive assessment of IBD. Further studies are needed incorporating AI use in US while also investigating its role in monitoring IBD disease activity. We hope this scoping review will serve as a future direction for subsequent research in this area.

**Funding Agencies:**

